# Pandemic

**DOI:** 10.3201/eid2210.160795

**Published:** 2016-10

**Authors:** Jennifer B. Nuzzo

**Affiliations:** University of Pittsburgh Medical Center/Center for Health Security, Baltimore, Maryland, USA

**Keywords:** Pandemic, epidemic, SARS, Ebola, MERS, West Nile virus, Zika, Lyme disease, cholera, antimicrobial resistance, contagion, viruses, bacteria

During the past 2 years, back-to-back epidemics of Ebola and Zika have stunned even seasoned public health officials with unprecedented levels of illness and death that have been associated with these events. Both viruses, previously thought to cause only limited outbreaks, surprised the people of the world with a seemingly sudden ability to spread across multiple countries and cause illness in new, previously unthinkable ways. As we struggle to understand both the scale and impact of the Zika and Ebola crises, we are forced to once again ask: how did this happen and what can we do to prevent the next one?

Given these events, Pandemic: Tracking Contagions, From Cholera to Ebola and Beyond by Sonia Shah makes for a very timely read. Although there have been several other books that have examined the origins of pandemics, Shah attempts to differentiate her analysis by using Rita Colwell’s description of the “Cholera Paradigm” as a framework for discussing the categories of social and environmental change; for example, locomotion, filth, crowds, and social dynamics that can explain how the microbes can emerge and spread disease around the world (*1*). In doing so, Pandemic takes its reader from the initial “spillover” of cholera from copepods in the Sundarbans wetlands of the Bay of Bengal through a series of historical outbreaks, including the recent outbreaks in Haiti.

While tracking cholera’s journey, Shah discusses other pathogens that have emerged and progressed down cholera’s path from isolated microorganism to pandemic pathogen: severe acute respiratory syndrome, Ebola, Middle East respiratory syndrome, West Nile, and Lyme disease, as well as pathogens that have acquired antimicrobial resistance. In doing so, Shah offers useful accounting of the history of the most consequential epidemic events that have occurred in the past 50 years. Shah’s personal experience in battling methicillin-resistant *Staphylococcus aureus* within her family serves as a particularly compelling illustration of how challenging even seemingly simple infections can be.

Scientists and practitioners who have been grappling with these diseases may quibble with Shah’s simplified explanations. The book’s central metaphor, the cholera paradigm, although somewhat instructive, is an imperfect model for explaining the rise and spread of pandemics. While some dimensions of cholera can be stretched to fit aspects of recent epidemics, there is scant evidence supporting how the central thesis of the cholera paradigm, that transmission can be in part explained by climate, applies to pandemics caused by other organisms, such as influenza. Additionally, that cholera today is largely a problem for countries without modern sanitation may create false assurances about all countries’ risks for pandemics.

Seasoned practitioners who know that there are almost no magic bullets in public health will also likely take issue with Shah’s discussion of potential ways to prevent and mitigate pandemics. Although real progress toward a pandemic-free world will likely require a long list of changes: some big, many of which are mundane, Shah’s attempts to offer up salvation in a single chapter comes across as more naive than inspiring.

Although Pandemic doesn’t offer a simple recipe for how to prevent the next pandemic, those who devote their careers to these issues should appreciate Shah’s attempt to elevate public understanding of these events. Rather than portray pandemics as freak, science-fiction–esque occurrences that can be quickly resolved and then forgotten, in its extensive discussion of the common conditions that create them, Shah illustrates why we should view pandemics as expected, recurring events and, therefore, should plan to invest in systemic, long-lasting preparedness. At a time when the current public debate about responding to Zika appears to be focused on the reallocation of Ebola funding despite the ongoing challenges posed by that not-yet-over epidemic, this is a message that is worth repeating.
